# Supplementary material for: 17β-Estradiol (E2) Activates Matrix Mineralization through Genomic/Nongenomic Pathways in MC3T3-E1 Cells
Source: Int J Mol Sci. 2024 Apr 26;25(9):4727. doi: 10.3390/ijms25094727 (PMC11083456; doi:10.3390/ijms25094727)
Supplement: Supplementary file 1 [file ijms-25-04727-s001.zip › SupS1 (revised).pdf]

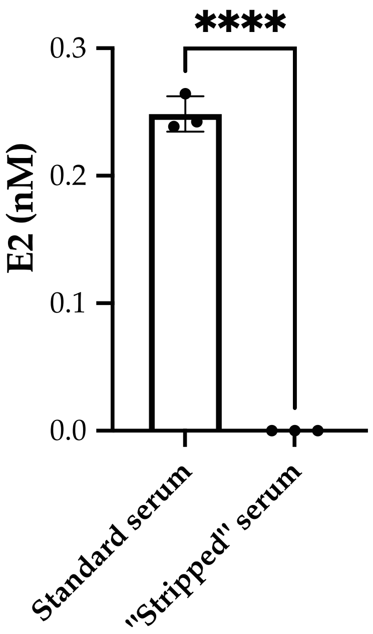

Supplementary Figure S1. E2 in standard fetal bovine serum (FBS) and stripped serum. E2 was measured by liquid chromatography with triple quad mass spectrometer (LC-MS/MS; LCMS-8050, Shimadzu). For E2 measurement, 17β-Estradiol-2, 4, 16, 16-d4 are added to serum sample as internal standards. Samples are pretreated using Isolute SLE+ prior to analysis and derivatized with 0.1 M Sodium bicarbonate and 1 mg/mL (acetone) Dansyl Chloride 1 mg/mL. Phase separation on a Acquity UPLC BEH C18 (1.7 μm× 2.1 ×50 mm) column (Waters) was performed using 0.1% formic acid in water and methanol, 60 ºC. The analytical measuring range (AMR) for E2 is over 0.07 nM. FBS is obtained from biowest and treated with/without "Stripping serum" method. Data presented as mean ± SD (n=3) are representative of at least three independent experiments. ****$P < 0.0001$ versus control (no E2) using unpaired t test.
