# Supplementary material for: 17β-Estradiol (E2) Activates Matrix Mineralization through Genomic/Nongenomic Pathways in MC3T3-E1 Cells
Source: Int J Mol Sci. 2024 Apr 26;25(9):4727. doi: 10.3390/ijms25094727 (PMC11083456; doi:10.3390/ijms25094727)
Supplement: Supplementary file 1 [file ijms-25-04727-s001.zip › SupS2(revised).pdf]

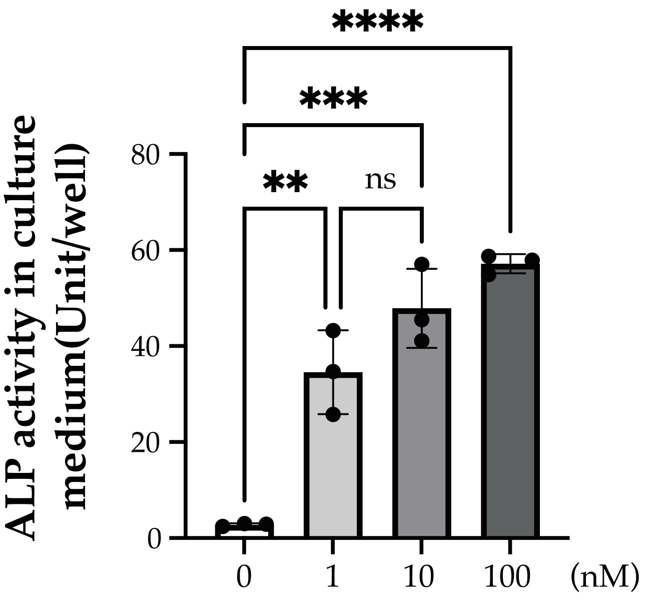

Supplementary Figure S2. The effect of E2 treatment on ALP activity of MC3T3-E1 in stripped FBS culture.

ALP assay. ALP activities in the culture medium at Day 9 of the cells stimulated with/without E2 (1-100 nM) at Day 0. Data presented as mean ± SD (n=3) are representative of at least three independent experiments. ***P* < 0.01, ****P* < 0.001 and *****P* < 0.0001 versus control (no E2) using Bonferroni' s test. ns: not significant.
