# Supplementary material for: 17β-Estradiol (E2) Activates Matrix Mineralization through Genomic/Nongenomic Pathways in MC3T3-E1 Cells
Source: Int J Mol Sci. 2024 Apr 26;25(9):4727. doi: 10.3390/ijms25094727 (PMC11083456; doi:10.3390/ijms25094727)
Supplement: Supplementary file 1 [file ijms-25-04727-s001.zip › SupS3 (reivsed).pdf]

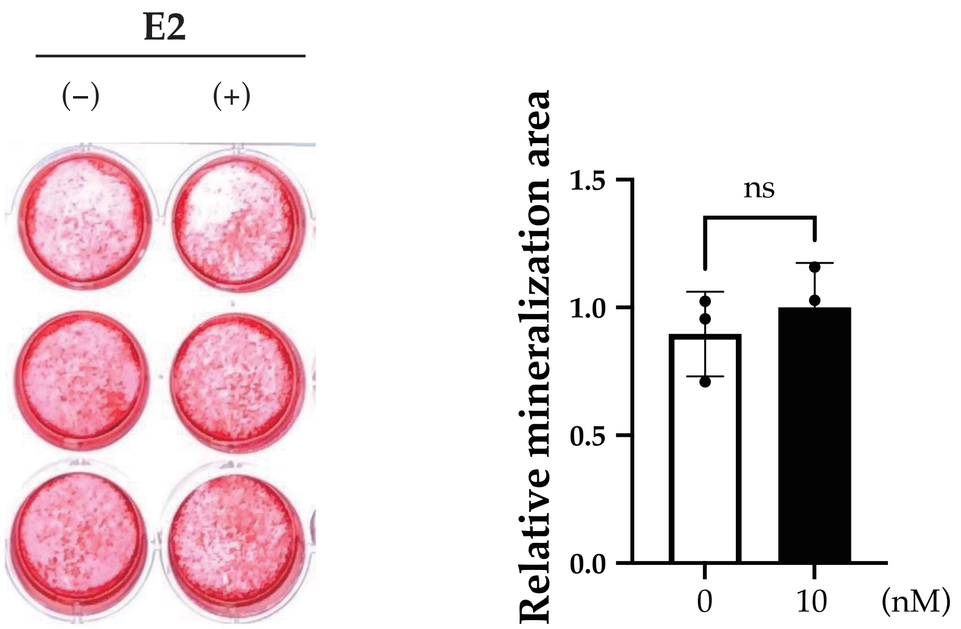

Supplementary Figure S3. The effect of E2 treatment on matrix mineralization of MC3T3-E1 in standard FBS culture.
Matrix mineralization assay. MC3T3-E1 were cultured in the media including standard FBS with AA/βGP and stimulated with or without E2 (10 nM) at Day 0. The cells were fixed with 10% formaldehyde and stained with Alizarin Red S at Day 12. Data presented as mean ± SD (n=3) are representative of at least three independent experiments. ns: not significant.ns, not significant.
